# Predictors of insufficient peak amikacin concentration in critically ill patients on extracorporeal membrane oxygenation

**DOI:** 10.1186/s13054-018-2122-x

**Published:** 2018-08-19

**Authors:** Cyril Touchard, Alexandra Aubry, Philippine Eloy, Nicolas Bréchot, Guillaume Lebreton, Guillaume Franchineau, Sebastien Besset, Guillaume Hékimian, Ania Nieszkowska, Pascal Leprince, Charles-Edouard Luyt, Alain Combes, Matthieu Schmidt

**Affiliations:** 1 Medical Intensive Care Unit, iCAN, Institute of Cardiometabolism and Nutrition, Hôpital de la Pitié–Salpêtrière, Assistance Publique–Hôpitaux de Paris, Sorbonne University , Paris 6, 47, bd de l’Hôpital, 75651 Paris Cedex 13, France; 2 Laboratory of Microbiology, Hôpital de la Pitié–Salpêtrière, Assistance Publique–Hôpitaux de Paris, Sorbonne University , Paris 6, 47, bd de l’Hôpital, 75651 Paris Cedex 13, France; 3 Department of Epidemiology, Biostatistics and Clinical Research, Bichat Hospital, AP-HP, F-75018 Paris, France; 4 Cardiac Surgery Department, iCAN, Institute of Cardiometabolism and Nutrition, Hôpital de la Pitié–Salpêtrière, Assistance Publique–Hôpitaux de Paris, Sorbonne University , Paris 6, 47, bd de l’Hôpital, 75651 Paris Cedex 13, France

**Keywords:** Amikacin, Pharmacokinetics, Acute respiratory distress syndrome, Cardiac failure, Shock, Volume of distribution

## Abstract

**Background:**

Amikacin infusion requires targeting a peak serum concentration (C_max_) 8–10 times the minimal inhibitory concentration, corresponding to a C_max_ of 60–80 mg/L for the least susceptible bacteria to theoretically prevent therapeutic failure. Because drug pharmacokinetics on extracorporeal membrane oxygenation (ECMO) are challenging, we undertook this study to assess the frequency of insufficient amikacin C_max_ in critically ill patients on ECMO and to identify relative risk factors.

**Methods:**

This was a prospective, observational, monocentric study in a university hospital. Patients on ECMO who received an amikacin loading dose for suspected Gram-negative infections were included. The amikacin loading dose of 25 mg/kg total body weight was administered intravenously and C_max_ was measured 30 min after the end of the infusion. Independent predicators of C_max_ < 60 mg/L after the first amikacin infusion were identified with mixed-model multivariable analyses. Various dosing simulations were performed to assess the probability of reaching 60 mg/L < C_max_ < 80 mg/L.

**Results:**

A total of 106 patients on venoarterial ECMO (VA-ECMO) (68%) or venovenous-ECMO (32%) were included. At inclusion, their median (1st; 3rd quartile) Sequential Organ-Failure Assessment score was 15 (12; 18) and 54 patients (51%) were on renal replacement therapy. Overall ICU mortality was 54%. C_max_ was < 60 mg/L in 41 patients (39%). Independent risk factors for amikacin under-dosing were body mass index (BMI) < 22 kg/m^2^ and a positive 24-h fluid balance. Using dosing simulation, increasing the amikacin dosing regimen to 30 mg/kg and 35 mg/kg of body weight when the 24-h fluid balance is positive and the BMI is ≥ 22 kg/m^2^ or < 22 kg/m^2^ (Table 3), respectively, would have potentially led to the therapeutic target being reached in 42% of patients while reducing under-dosing to 23% of patients.

**Conclusions:**

ECMO-treated patients were under-dosed for amikacin in one third of cases. Increasing the dose to 35 mg/kg of body weight in low-BMI patients and those with positive 24-h fluid balance on ECMO to reach adequate targeted concentrations should be investigated.

**Electronic supplementary material:**

The online version of this article (10.1186/s13054-018-2122-x) contains supplementary material, which is available to authorized users.

## Background

Over the last decade, a growing number of patients have benefited from extracorporeal membrane oxygenation (ECMO) as rescue therapy for patients suffering from severe acute respiratory distress syndrome (ARDS) or refractory cardiogenic shock [1]. More than half of them will require antibiotic therapy during their ECMO run to treat a primary infection or secondary acquired infection [2]. Because little is known about the impact of ECMO on antibiotic pharmacokinetics (PK), antibiotic administration on ECMO remains a challenge. Although suboptimal antibiotic dosing in this complex group of patients may have fatal consequences, available data are actually limited to animal [3], simulated ex-vivo [4], or small retrospective human studies [5]. Sequestration in the circuit, increased volumes of distribution (Vd), and decreased drug clearance have been implicated to explain antibiotic–PK modifications in that context [6]. Moreover, the underlying disease in these extremely severely ill patients may also be key. Empirical combination antibiotic therapy, aimed at covering Gram-negative bacilli, usually combines a β-lactam and an aminoglycoside [7]. Considering its good bactericidal activity against *Pseudomonas aeruginosa* and its low resistance rate observed with other Gram-negative bacilli, amikacin is frequently used in that context [8]. Its antibacterial effect is determined by the ratio of peak serum concentration (C_max_) to the targeted pathogen’s minimal inhibitory concentration (MIC), with optimal antibacterial activity obtained with a C_max_/MIC ratio of 8–10. Consequently, the amikacin C_max_ range should be 64–80 mg/L [9], while the most recent French guidelines recommended C_max_ > 60 mg/L [10]. Despite administering an amikacin dose of 25 mg/kg total body weight (TBW), only 67–72% of intensive care unit (ICU) patients have been found to achieve that objective [11, 12]. In this setting, further PK changes in ECMO-treated patients would be anticipated. Therefore, this open-label, monocentric, prospective study was designed to determine the frequency and identify factors predictive of insufficient amikacin C_max_ in critically ill patients, and to analyze the probability of attaining the established PK target (i.e. greater than 60 mg/L but below 80 mg/L).

## Methods

### Setting

This prospective observational study was conducted in a 26-bed medical–surgical ICU at a university hospital. Its protocol was in accordance with the ethical standards of our hospital’s Institutional Review Board (Committee for the Protection of Human Subjects). In accordance with French law, informed consent was not obtained for demographic, physiological and hospital-outcome data analyses because this observational study did not modify existing diagnostic or therapeutic strategies. However, patients and/or relatives were informed about the anonymous data collection and that they could decline inclusion. This database was registered at the Commission Nationale l’Informatique et des Libertés (CNIL, registration number 1950673).

### Study design and patients

We prospectively included all consecutive patients, from January 2015 through February 2016, who received an intravenous amikacin loading dose for suspected Gram-negative infection in a context of venoarterial (VA) or venovenous (VV) ECMO in our ICU. Only the first dose of amikacin was studied. Four types of ECMO systems with poly-4-methyl-1-pentene membrane oxygenators (PLS system, Cardiohelp system, both Maquet, Rastatt, Germany; Hilite 7000 LT system, Medos, Stolberg, Germany; ECC.05 system, Sorin, Mirandola, Italy) were used during the study period. Exclusion criteria were (1) incorrect amikacin regimen (< 23 or > 27 mg/kg TBW); (2) incorrect time of amikacin infusion (± 5 min); or (3) incorrect time (± 5 min) or absence of C_max_ determination. Patients with incorrect time of trough serum concentration (C_min_) determination (± 1 h) were not excluded if C_max_ had been obtained correctly.

### Amikacin administration and serum-concentration dosage

Amikacin was administered according to our ICU’s written standardized protocol: 25 mg/kg TBW (weighing bed weight of the day), diluted in 50 mL of 0.9% NaCl and continuously infused over 30 min. When the patient’s weight was > 120 kg, a maximum of 120 kg was considered to calculate the loading amikacin dose (five patients in our study). C_max_ was assessed 30 min after that infusion ended and C_min_ 24 h after the latter. Our Microbiology Laboratory used a fluorescence-polarization immunoassay to determine amikacin concentrations, as a routine procedure available 24 h/day, 7 days/week [13].

### Data collection

Demographic data, Simplified Acute Physiology Score (SAPS) II [14], and reason for ECMO were collected for all patients. ECMO-membrane duration was defined as the delay between membrane first use and the time of amikacin infusion. Organ dysfunction at inclusion (i.e. the day of amikacin administration) was assessed with the Sequential Organ-Failure Assessment (SOFA) score. In addition, laboratory tests, including coagulation parameters, complete blood count, electrolytes, liver enzymes, urea, creatinine, and bilirubin, were run. At inclusion, the inotrope score was calculated [15], defined as:

Dobutamine dose (γ/kg/min) + [Norepinephrine dose (γ/kg/min) + Epinephrine dose (γ/kg/min)] × 100.

The 24-h fluid balance was defined as the difference between fluid intake and fluid output over the 24 h before amikacin infusion. Proteinemia and hematocrit changes during the 24 h preceding inclusion were calculated as follows:

(X_0h_ – X_24h_)/(X_0h_ + X_24h_)/2.

In addition, renal function was characterized according to the Kidney Disease: Improving Global Outcomes (KDIGO) classification [16]. Acute renal failure in ICU survivors was defined as KDIGO ≥ 2. Lastly, infection sites and identified pathogens were noted.

### Statistical analyses

Categorical variables are expressed as number (percentage) and compared using the chi-square test. Continuous variables are expressed as medians (1st; 3rd quartile) and compared using Student’s *t* test or Wilcoxon’s signed-rank test. Pre-amikacin infusion factors associated with a C_max_ < 60 mg/L or a C_max_ > 80 mg/L were selected using univariable mixed models. Demographics and clinical and biological factors with a *p* value ≤0.10 in our univariable analyses were entered into the multivariable mixed model. The list of data entered in both models is provided in Additional file 1: Table S1. Few relevant variables with a plausible clinical link despite a value of *p* > 0.10 in the univariate analysis were forced into the models (Additional file 1: Table S1). Continuous variables were transformed into categorical variables (the optimal thresholds were defined by analyzing mortality in each corresponding variable quartile). Thereafter, multiple backward-stepwise logistic-regression analyses eliminated variables with an exit threshold set at *p* > 0.05. All potential explanatory variables included in the multivariable analyses were subjected to collinearity analysis with a correlation matrix. In the case of collinearity between factors, the most clinically relevant factor was chosen to construct the multivariable model. Results are reported as odds ratios (ORs) and their 95% confidence intervals (CIs).

Given the observed C_max_ and the administered dose/kilogram, we assumed amikacin PK linearity [17–20] to simulate individual C_max_ for the following dosing regimen: 20 mg/kg, 25 mg/kg, 30 mg/kg, 35 mg/kg TBW. In order to provide dosing regimen recommendations, for each dosing regimen, we estimated the probability of C_max_ in the therapeutic range (60–80 mg/L) for the different categories of the risk factors found predictive of under-dosing in the multivariable analysis.

Analyses were computed using StatView v5.0 (SAS Institute, Cary, NC, USA), SPSS v22.0 software (SPSS, Chicago, IL, USA) and R v3.3.0 (http://www.R-project.org).

## Results

### Study population

**Fig. 1:**
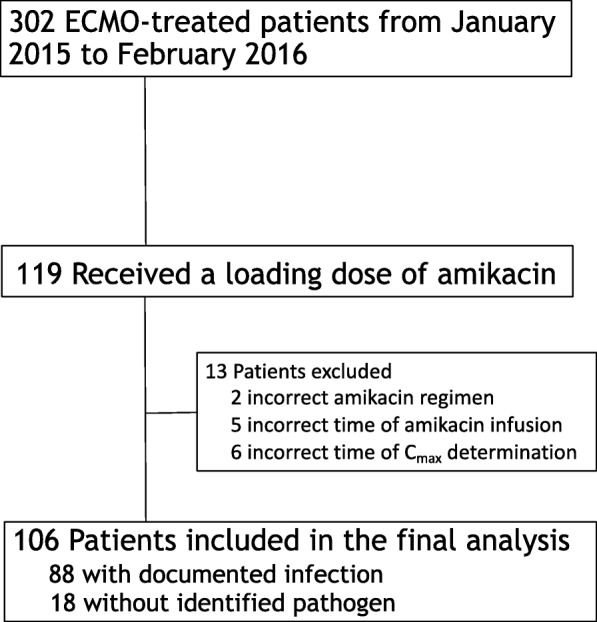
Study flow chart. ECMO, extracorporeal membrane oxygenation; C_max,_ peak serum concentration

During the study period, among 302 ECMO-treated patients in our ICU, 119 (39%) received an amikacin loading dose for severe sepsis or septic shock; 13 were excluded from our analysis for the main reasons listed in Fig. 1. Consequently, PK of a first amikacin loading dose on ECMO were studied in 106 patients. Patient characteristics at ICU admission and study inclusion are shown in Table 1. Median (IQR) age was 55 (45; 62) years, while median SAPS II was 68 (47; 81). VA-ECMO supported 68% of the patients. On the day of amikacin infusion, the SOFA score was 15 (12; 18) with 54 (51%) patients on renal replacement therapy. The main infection sites were lung and cannula for, respectively, 77% and 14% patients, with associated bacteremia in 15 (17%); the most frequently identified pathogens were *P. aeruginosa, Enterobacter* spp. and *Escherichia coli* (Additional file 1: Table S2).

**Table 1:** Total population characteristics and univariable analyses of factors predictive of amikacin C_max_ < 60 mg/L

Characteristic	Total population (*n* = 106)	C_max_ < 60 mg/L (*n* = 41)	C_max_ ≥ 60 mg/L (*n* = 65)	*p*
At ICU admission				
Age, years	55 (44–62)^a^	54 (39–64)	57 (48–60)	0.61
Male	77 (73)	28 (68)	49 (75)	0.42
SAPS II	68 (47; 81)	70 (46; 81)	68 (47; 81)	0.66
BMI*,* kg/m^2^	26 (23; 31)	25 (22; 29)	26 (24; 33)	0.03
At inclusion				
SOFA score	15 (12; 18)	16 (13; 18)	14 (11; 18)	0.12
Weight, kg	80 (71; 97)	79 (65; 91)	80 (73; 98)	0.24
Height, m	1.72 (1.65–1.78)	1.70 (1.65–1.75)	1.75 (1.68–1.78)	0.18
ICU admission-to-ECMO interval, days	1 (0; 7)	1 (0; 5)	1 (0; 8)	0.52
ICU admission-to-C_max_ interval, days	9 (5; 15)	8 (3; 15)	9 (6; 15)	0.46
ECMO-to-C_max_ interval, days	6 (3; 11)	7 (1; 12)	6 (3; 9)	0.99
Inotrope score, μg/kg/min	30 (5; 131)	68 (10; 150)	21 (5; 105)	0.12
Reason for ECMO				0.68
Cardiogenic shock	49 (46)	17 (41)	32 (49)	
Post-cardiac transplant	9 (8)	3 (7)	6 (9)	
Cardiac arrest	7 (7)	2 (5)	5 (8)	
Post-cardiotomy	6 (6)	2 (5)	4 (6)	
Severe ARDS	35 (33)	17 (41)	18 (28)	
VA-ECMO	72 (68)	25 (61)	47 (72)	0.22
ECMO flow, L/min	4.0 (3.0; 5.0)	4.2 (3.2; 5.4)	3.7 (2.9; 4.6)	0.13
Weight-indexed ECMO flow, L/min/kg	0.49 (0.37–0.58)	0.53 (0.42–0.64)	0.46 (0.36–0.55)	0.013
ECMO-membrane duration, days	5 (2; 8)	4 (1; 8)	5 (3; 8)	0.16
Laboratory finding				
Aspartate aminotransferase, mmol/L	79 (41; 267)	99 (44; 747)	71 (39; 188)	0.08
Alanine aminotransferase, mmol/L	56 (27; 157)	69 (41; 377)	53 (25; 126)	0.08
Bilirubin, mmol/L	27 (16; 67)	23 (17; 66)	30 (15; 67)	0.92
Prothrombin time, %	65 (51; 77)	62 (43; 76)	66 (54; 78)	0.16
V factor, %	75 (47; 112)	59 (29; 107)	83 (57; 123)	0.08
Proteinemia, g/L	53 (43; 58)	49 (44; 55)	55 (48; 59)	0.02
Albuminemia, g/L	21 (19; 25)	22 (19; 25)	21 (19; 25)	0.86
Prealbuminemia, g/L	0.13 (0.09; 0.19)	0.13 (0.11; 0.19)	0.13 (0.09; 0.19)	0.65
Hematocrit, %	25 (23; 27)	24 (22; 27)	25 (23; 29)	0.09
Lactates, mmol/L	1.8 (1.2; 4.0)	1.9 (1.1; 6.3)	1.8 (1.3; 3.8)	0.89
Hemodilution parameter				
24-h fluid balance, mL	225 (− 980; 1607)	1000 (200; 2045)	− 371 (− 1564; 1342)	< 0.001
24-h protidemia delta, %	0 (− 5; 3.5)	0 (− 5.6; 1.8)	0 (− 3.9; 5.0)	0.37
24-h hematocrit delta, %	0.0 (− 5.2; 3.5)	0.0 (− 5.6; 1.8)	0.0 (− 3.9; 5.0)	0.38
GFR, mL/min	2 (0; 69)	0 (0; 84)	6 (0; 61)	0.97
Renal function				0.42
KDIGO-0	30 (28)	14 (34)	16 (25)	
KDIGO-1	11 (10)	2 (5)	9 (14)	
KDIGO-2	6 (6)	2 (5)	4 (6)	
KDIGO-3	59 (56)	23 (56)	36 (55)	
KDIGO ≥2	65 (61)	25 (61)	40 (62)	0.95
Dialysis	9 (8)	1 (2)	8 (12)	0.07
CRRT	45 (42)	22 (54)	23 (35)	0.06
Outcome				
ICU mortality	57 (54)	21 (51)	36 (55)	0.67
Hospital mortality	58 (55)	21 (51)	37 (57)	0.57
ECMO duration, days	18 (10; 26)	18 (8.5; 24.5)	18 (10; 27)	0.83
Mechanical ventilation duration, days	22 (12; 41)	26 (17; 46)	20 (12; 38)	0.32
RRT duration after C_max_, days	15 (5; 24)	15 (4; 26)	14 (5; 21)	0.64
AKI^b^ at ICU discharge in survivors^c^	11 (23)	5/20 (12)	6/29 (9)	0.65

*AKI* acute kidney injury, *ARDS* acute respiratory distress syndrome, *BMI* body mass index, *C*_*max*_ peak serum concentration *CRRT* continuous renal replacement therapy, *GFR* glomerular filtration rate, *ICU* intensive care unit, *KDIGO* Kidney Disease: Improving Global Outcomes, *SAPS* Simplified Acute Physiology Score, *SOFA* Sequential Organ-Failure Assessment, *VA-ECMO* venoarterial extracorporeal membrane oxygenation

^a^Values are expressed as median (1st; 3rd quartile) or number (percentage)

^b^Defined as KDIGO ≥ 2

^c^Based on 49 ICU survivors

### Pharmacokinetic parameters

Amikacin PK parameters are reported in Table 2. The median ECMO cannulation-to-C_max_ interval was 9 (5; 15) days. After receiving a loading dose of 25 (24; 25.5) mg/kg TBW, median C_max_ was 65.8 (51.8; 82.4) mg/L. C_max_ was < 60 mg/L in 41 patients (39%) and > 80 mg/L in 27 (25%) (Additional file 1: Figure S1**)**. Median C_min_ for 85 patients was 7.25 (3.6; 13.6) mg/L, with only 24 patients (28%) having a value < 2.5 mg/L.

**Table 2:** Pharmacokinetic/pharmacodynamic parameters at inclusion in 106 patients

Variable	Value^a^
Weight at time of C_max_, kg	80.0 (71.0; 97.5)
Amikacin dose, mg	2000 (1750; 2500)
Amikacin regimen, mg/kg	25.0 (24.0; 25.5)
Amikacin C_max_, mg/L	65.8 (51.8; 82.4)
Patients with C_max_ < 60 mg/L	41 (39)
Patients with C_max_ > 80 mg/L	27 (25)
ICU admission-to-C_min_ interval, h	24.1 (23.4; 24.8)
Amikacin C_min_, mg/L	7.25 (3.60; 13.60)
Patients with C_min_ < 2.5 mg/L^b^	24 (28)

C_max_ peak serum concentration, *C*_*min*_ trough serum concentration

^a^Values are expressed as median (1st; 3rd quartile]) or number (percentage)

^b^Available for 85 patients

### Factors predictive of C_max_ < 60 mg/L

**Fig. 2:**
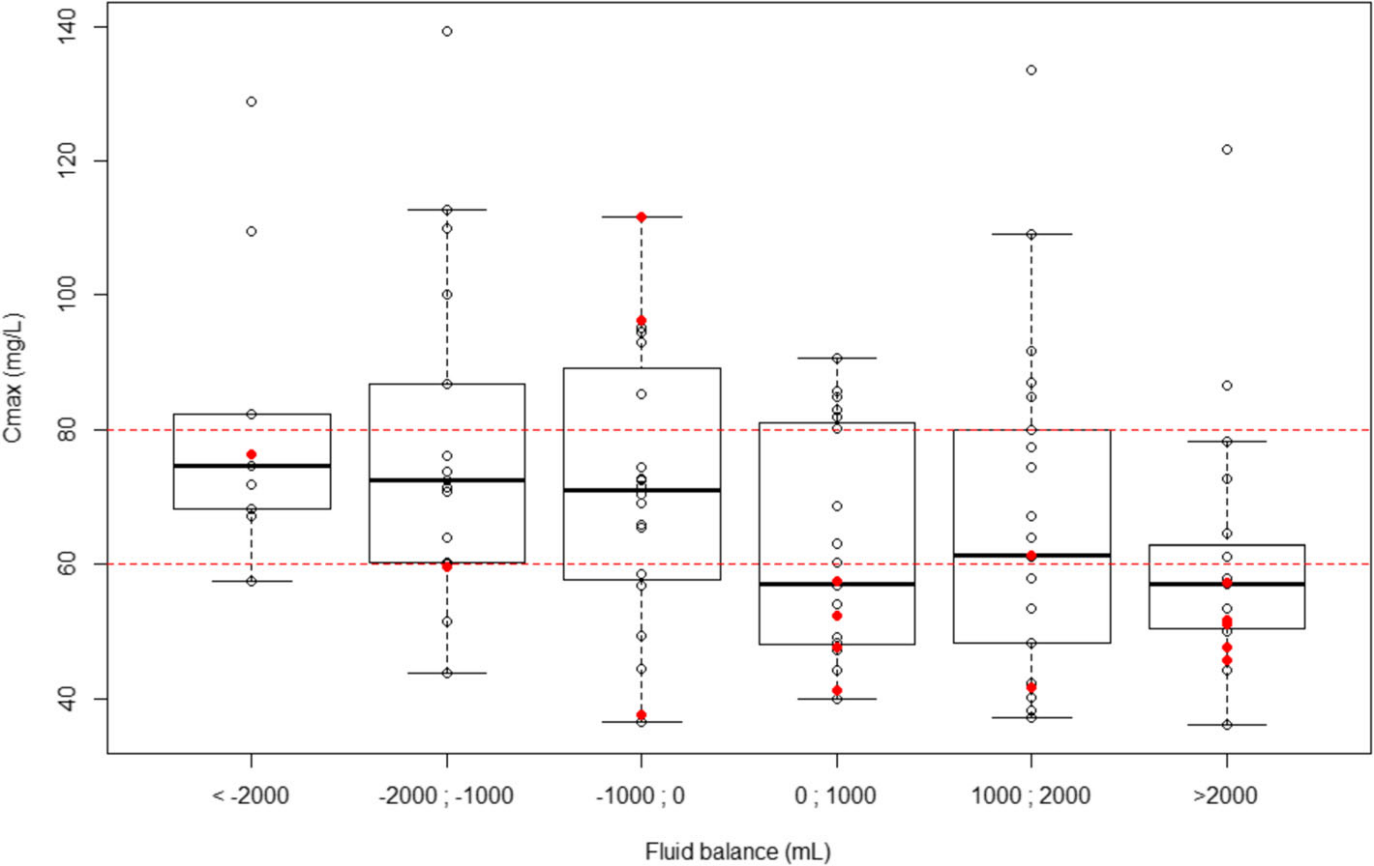
Amikacin peak serum concentration (C_max_) after a single dose of 25 mg/kg total body weight according to 24-h fluid balance on extracorporeal membrane oxygenation (ECMO). Concentrations in patients with body mass index (BMI) < 22 kg/m^2^ or > 22 kg/m^2^ are represented by red dots and circles, respectively. Boxplots represent the distribution of the concentrations. The lower and upper borders correspond to the first and third quartiles. The upper whisker extends from the borders to the highest value that is within 1.5 * interquartile range (IQR) of the borders, or the distance between the first and third quartiles. The lower whisker extends from the borders to the lowest value within 1.5 * IQR of the hinge. Red dashed lines represent the therapeutic margin (60–80 mg/L)

Univariable analyses selected lower body mass index (BMI), higher liver-enzyme concentrations, lower proteinemia, lower hematocrit and positive 24-h fluid balance as factors predictive of C_max_ < 60 mg/L (Table 1). Multivariable analyses retained BMI < 22 kg/m^2^ (OR, 6.38; 95% CI, 1.79–22.77; *p =* 0.043) and 24-h fluid balance (OR per 500-mL increment, 1.28; 95% CI, 1.05–1.65; *p =* 0.041) as being independently associated with a higher risk of C_max_ < 60 mg/L. A linear relationship between the probability of amikacin C_max_ < 60 mg/L and 24-h fluid balance was observed. That probability reached > 60% when 24-h fluid balance exceeded 2000 mL (Fig. 2). Notably, we did not find any significant association between C_max_ < 60 mg/L and ECMO settings (i.e. VA or VV), ECMO flow, and ECMO membrane duration.

### Factors predictive of C_max_ > 80 mg/L

Univariable analyses selected higher BMI, ECMO flow, dialysis, and higher hematocrit as factors predictive of C_max_ > 80 mg/L **(**Additional file 1: Table S3**)**: 24-h-hour fluid balance was forced into the logistic regression (Additional file 1: Table S1**)**. Multivariable analyses retained only higher BMI (OR, 1.10; 95% CI, 1.03–1.18; *p =* 0.0037) as being independently associated with a higher risk of C_max_ > 80 mg/L.

### Dosing simulations

**Fig. 3:**
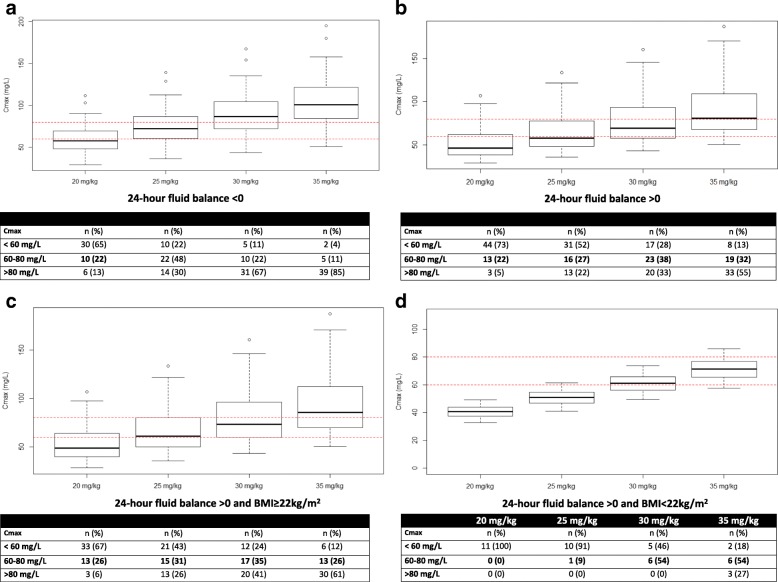
Simulated peak serum concentration (C_max_) and probability of amikacin efficacy, under-dosing, and overdosing for various dosing regimens in a critically ill patient on extracorporeal membrane oxygenation (ECMO) with a negative 24-h fluid balance (**a**); a positive 24-h fluid balance (**b**); a positive 24-h fluid balance and body mass index (BMI) ≥ 22 kg/m^2^ (**c**); or a positive 24-h fluid balance and BMI < 22 kg/m^2^ (**d**)

Figure 3 describes the simulated C_max_ for various amikacin dosing regimens in a critically ill patient on ECMO with either a 24-h positive or negative fluid balance and a BMI < or ≥ 22 kg/m^2^. The probability of target attainment (C_max_ 60–80 mg/L), under-dosing (C_max_ < 60 mg/L) and overdosing (C_max_ > 80 mg/L) is also reported. These data show that increasing amikacin doses up to 30 mg/kg for patient with a positive 24 h-fluid balance may increase the likelihood of reducing under-dosing. Based on dosing simulation, 28% of these patients still exhibit under-dosing with 30 mg/kg, whereas it will also expose the patient to more frequent overdosing **(**Fig. 3). In addition, increasing amikacin dosing regimen up to 30 mg/kg and 35 mg/kg TBW when 24-h fluid balance is positive and the BMI is ≥ 22 kg/m^2^ or < 22 kg/m^2^ (Table 3), respectively, would have potentially led to the therapeutic target being reached in 42% of patients while reducing under-dosing incidence to 23% of patients (Additional file 1: Figure S2).

**Table 3:** Proposal for amikacin dosing regimens on ECMO according to 24-h fluid balance and body mass index (BMI)

	BMI < 22 kg/m^2^	BMI ≥ 22 kg/m^2^
Negative 24-h fluid balance	25 mg/kg	25 mg/kg
Positive 24-h fluid balance	35 mg/kg^a^	30 mg/kg

^a^Based on only 11 patients with these characteristics

### Outcomes

Overall in-ICU mortality was 54% after, respectively, 22 (12; 41) days on mechanical ventilation and 18 (10; 26) days on ECMO. Among ICU survivors, the 11 patients (23%) who suffered acute kidney injury (AKI) had comparable C_max_ levels to those without AKI (Table 2). No association was found between ICU mortality and insufficient C_max_ or too high C_min_. In addition, ICU mortality did not differ significantly in patients with C_max_ < 60, 60–80, or > 80 mg/L (59%, 52%, or 51%, respectively).

## Discussion

The results of our large prospective study indicate that amikacin under-dosing on ECMO after a loading dose of 25 mg/kg TBW is frequent, with more than a third of the patients having C_max_ < 60 mg/L. In addition, using a 25 mg/kg amikacin dosing regimen led to 64% of patients with a C_max_ outside the targeted range of 60–80 mg/L. Independent risk factors for amikacin under-dosing were BMI < 22 kg/m^2^ and positive 24-h fluid balance, in agreement with previous studies without ECMO [17–20]. No association between under-dosing or overdosing of amikacin and outcome was found. Last, dosing simulations might suggest to increase the amikacin dosing regimen up to 30 mg/kg and 35 mg/kg TBW when 24-h fluid balance is positive and the BMI is > 22 kg/m^2^ or the BMI is < 22 kg/m^2^, respectively.

Describing antibiotic PK in the population on ECMO is essential to understand whether special dosing may be required. Unfortunately, such data on ECMO-treated adults are sparse and the available literature is mainly restricted to ex-vivo studies [4, 6, 21]. Systemic inflammatory responses, organ dysfunction, drug interactions and organ support are all known to affect antibiotic PK in critically ill patients. Drug PK seems to be more markedly modified on ECMO [6]. General PK parameters of hydrophilic antibiotics, like aminoglycosides, are low Vd, predominantly with renal clearance, and low intracellular penetration [22].

Recently, in a large case series of 146 severely ill patients receiving 181 amikacin infusions, including 15% on ECMO, C_max_ was < 60 mg/L during 33% of the episodes. Independent risk factors were BMI < 25 kg/m^2^ and positive 24-h fluid balance [11]. Although many factors, e.g. the extracorporeal circuit, high-volume fluid infusion and the extreme severity of critical illness in the patients could contribute to a larger Vd on ECMO, we found similar frequencies of amikacin under-dosing and overdosing with the same risk factors. Based on a smaller cohort of 46 ECMO-treated patients, Gelisse et al. reported a similar percentage of C_max_ insufficiency, when matched with critically ill patients without ECMO support [5]. We postulate that, as demonstrated without ECMO [19–21], the intensity of interstitial fluid shift during sepsis on ECMO will result in a larger Vd, which might lower plasma antibiotic concentrations. It is worth noting that the increased Vd in septic patients may be attributed to hypoalbuminemia and the resulting decreased oncotic pressure [22], which was low in our population.

Low BMI (i.e. < 22 kg/m^2^) was the second independent risk factor for C_max_ < 60 mg/L, which agrees with published observations [11, 12]. With hydrophilic drugs, like amikacin, using TBW for dose calculation in high-BMI patients, instead of ideal body weight or adjusted body weight, could overestimate amikacin Vd. Indeed, higher BMI was the only factor that was independently associated with a higher risk of overdosing in our study. Surprisingly, although, a poor TBW–Vd relationship has been thoroughly described, the amikacin loading dose is still commonly adjusted to TBW. Therefore, we cannot rule out the fact that using adjusted body weight in obese patients [23] would have led to different findings.

Notably, Allou et al. [24] and Galvez et al. [25] reported, without a clear explanation, that amikacin overexposure (i.e. C_max_ > 80 mg/L) might potentially be associated with increased mortality. Although, our study was not designed to analyze mortality, we did not observe any C_max_–mortality association. Moreover, an amikacin C_min_ > 2.5 mg/L was not significantly associated with a higher AKI rate at ICU discharge. Considering the high amikacin under-dosing frequency in our ECMO-treated patients and our dosing simulations, our dosing simulation suggests that increasing the loading dose up to a 35 mg/kg TBW dose for low-BMI patients and those with positive 24-h fluid balance on ECMO could help to obtain adequate target concentrations. However, these dosing simulations deserve to be confirmed by future prospective studies or Monte-Carlo-based simulations. Less frequent under-dosing has also been reported without a higher AKI rate, using a higher loading dose [24, 26]. However, despite the current trend toward increasing antibiotic doses in ICUs, no beneficial effect on outcome has been clearly demonstrated. In addition, it can be argued that a lower amikacin C_max_ might be enough to obtain C_max_/MIC > 8 for most isolated strains that often have amikacin MICs ≤ 4 mg/L [26].

Our study’s strengths include the large cohort investigated, focusing on the impact of ECMO, its longitudinal design and a short interval retained for the correct time of C_max_ determination. However, it also has limitations. First, it is a single-center study in an ICU with a high volume of ECMO cases in a tertiary-care university hospital, and our results may not be generalizable to all ICUs. Second, our ECMO population was a mix of patients who received VA-ECMO and VV-ECMO support, with different outcomes and underlying illnesses. Given that the underlying diseases of these extremely ill patients is key in antibiotic PK modifications on ECMO, our results might have been different if we had focused on a subpopulation of patients on ECMO (i.e. those with cardiac or respiratory failure). However, our multivariable analysis did not identify reasons for ECMO and type of ECMO hook-up as factors predictive of C_max_ < 60 mg/L. Third, we cannot exclude that coadministration with another antibiotic and other ICU treatments might also have contributed to AKI at ICU discharge. Fourth, our dosing simulation was based on amikacin PK linearity [17–20] and a limited number of patients (*n* = 11) with positive 24-h fluid balance and BMI < 22 kg/m^2^. And finally, we cannot rule out that a portion of amikacin might have been sequestrated on the ECMO membrane, as previously described on the hemofilter membrane [27].

## Conclusions

Despite an amikacin loading dose of 25 mg/kg TBW in the ECMO-treated patients included in our large prospective study, we found that more than a third of them did not reach the targeted C_max_ > 60 mg/L. Increasing the dose to 35 mg/kg in low-BMI patients and those with positive 24-h fluid balance on ECMO to reach adequate targeted concentrations should be investigated. However, the impact of a higher amikacin loading dose on AKI frequency and outcomes remains unclear. Further human PK studies are urgently needed to confirm our results and develop population PK models to provide dosing guidelines for patients on ECMO.

## Additional file

Additional file 1: **Table S1.** List of the data included into the multivariable mixed models. **Table S2.** Infection sites, pathogens identified and their ECOFF for Amikacin in 88 patients with documented infections. **Figure S1.** Distribution of amikacin C_max_ concentrations in ECMO-treated patients. **Table S3.** Total population characteristics and univariable analyses of factors predictive of amikacin C_max_ > 80 mg/L. **Figure S2.** Amikacin C_max_ distribution within our population on ECMO with 25 mg/kg for all patients or with using potentially adapted dosing regimens based on 24-h fluid balance and BMI, as described in Table 3. (DOCX 273 kb)

## Abbreviations

AKI: Acute kidney injury
ARDS: Acute respiratory distress syndrome
BMI: Body mass index
C_max_: Peak serum concentration
C_min_: Trough serum concentration
ECMO: Extracorporeal membrane oxygenation
ICU: Intensive care unit
KDIGO: Kidney Disease: Improving Global Outcomes
MIC: Minimal inhibitory concentration
OR: Odds ratio
PK: Pharmacokinetics
SAPS: Simplified Acute Physiology Score
SOFA: Sequential Organ-Failure Assessment
TBW: Total body weight
VA-ECMO: Venoarterial extracorporeal membrane oxygenation
Vd: Volumes of distribution
