# Supplementary material for: Predictors of insufficient peak amikacin concentration in critically ill patients on extracorporeal membrane oxygenation
Source: Crit Care. 2018 Aug 19;22:199. doi: 10.1186/s13054-018-2122-x (PMC6098833; doi:10.1186/s13054-018-2122-x)
Supplement: Supplementary file 1 — Table S1. List of the data included into the multivariable mixed models. Table S2. Infection sites, pathogens identified and their ECOFF for Amikacin in 88 patients with documented infections. Figure S1. Distribution of amikacin Cmax concentrations in ECMO-treated patients. Table S3. Total population characteristics and univariable analyses of factors predictive of amikacin Cmax > 80 mg/L. Figure S2. Amikacin Cmax distribution within our population on ECMO with 25 mg/kg for all patients or with using potentially adapted dosing regimens based on 24-h fluid balance and BMI, as described in Table 3. (DOCX 273 kb) [file 13054_2018_2122_MOESM1_ESM.docx]

**Additional files**

**Predictors of insufficient peak amikacin concentration in critically ill patients under extracorporeal membrane oxygenation**

Cyril Touchard, MD^1^; Alexandra Aubry, MD, PhD^2^; Philippine Eloy, PharmD^3^ ; Nicolas Bréchot, MD, PhD^1^; Guillaume Lebreton, MD, PhD^4^; Guillaume Franchineau, MD^1^; Sebastien Besset, MD^1^; Guillaume Hékimian, MD^1^; Ania Nieszkowska, MD^1^; Pascal Leprince, MD, PhD^4^; Charles-Edouard Luyt, MD, PhD^1^; Alain Combes, MD, PhD^1^; Matthieu Schmidt MD, PhD^1^

.

**Additional File 1. List of the data included into the multivariable mixed models.**

| **Pre-amikacin infusion factors associated with** | |
| --- | --- |
| **C_max_ < 60 mg/L** | **C_max_ > 80 mg/L** |
| *Variables with* *p ≤ 0.10 in univariable analysis* | |
| - BMI<22kg/m^2^ | - BMI |
| - Proteinemia | - ECMO flow |
| - Hematocrit, | - Hematocrit |
| - 24-hour fluid balance | - Dialysis |
| - CRRT |  |
| - Weight indexed ECMO flow |  |
| *Variables forced into the model* | |
| - VA-ECMO | - 24-hour fluid balance |
| - ECMO-membrane duration |  |

BMI = body mass index, CRRT = continuous renal replacement therapy,

VA-ECMO = venoarterial extracorporeal membrane oxygenation

**Additional file 2. Infection Sites, Pathogens Identified and their ECOFF for Amikacin in 88 Patients with Documented Infections**

| **Parameter** | **Value** | **ECOFF^b^ Eucast for Amikacin (mg/L)** |
| --- | --- | --- |
| **Site, *n* (%)** |  |  |
| Ventilator-associated pneumonia | 68 (77) |  |
| Cannula infection | 12 (14) |  |
| Bloodstream infection | 15 (17) |  |
| **Microorganism, *n* (%)** |  |  |
| *Pseudomonas aeruginosa* | 29 (33) | 16 |
| *Enterobacter* spp. | 18 (20) | 8 |
| *Escherichia coli* | 14 (16) | 8 |
| *Klebsiella* spp. | 10 (11) | 8 |
| *Polymicrobial^a^* | 6 (7) | ND |
| *Enterococcus* spp*.* | 5 (6) | ND |
| *Proteus mirabilis* | 4 (4) | 8 |
| *Serratia marcescens* | 4 (4) | 8 |
| *Hafnia alvei* | 3 (3) | ND |
| *Staphylococcus aureus* | 3 (3) | 8 |
| *Staphylococcus epidermidis* | 3 (3) | ND |
| *Citrobacter* spp. | 2 (2) | 8 |
| *Haemophilus influenzae* | 2 (2) | 16 |
| *Streptococcus* spp. | 2 (2) | ND |
| *Neisseria* spp. | 1 (1) | ND |

^a^Includes ≥ 2 oropharyngeal pathogens.

^b^Epidemiological cut-off value (ECOFF): MIC value identifying the upper limit of the wild type population. A microorganism is defined as wild type (WT) for a species by the absence of acquired and mutational mechanisms of resistance to the agent.

**Additional file 3. Distribution of amikacin C_max_ concentrations in ECMO-treated patients.**


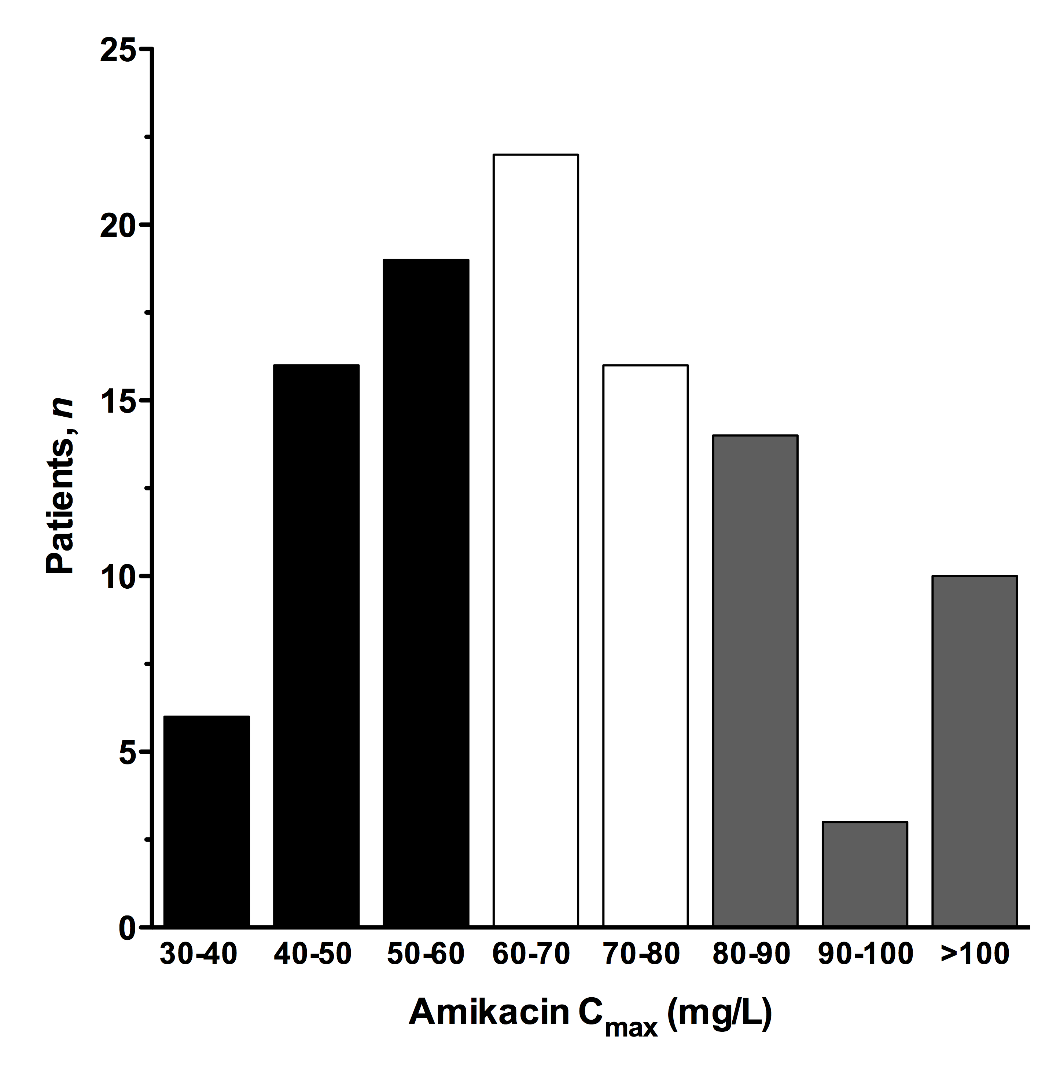


Black: underdosed; white: adequately dosed; grey overdosed. C_max,_ peak serum concentration; ECMO, extracorporeal membrane oxygenation.

**Additional file 4: Total Population Characteristics and Univariable Analyses of Factors Predictive of Amikacin C_max_ > 80 mg/L**

| **Characteristic** ^a^ | **C_max_ ≤ 80 mg/L**  **(*n* = 79)** | **C_max_ > 80 mg/L**  **(*n* = 27)** | ***p*** |
| --- | --- | --- | --- |
| At ICU admission |  |  |  |
| Age, years | 54 (41–62) | 57 (50–64) | 0.17 |
| Males | 60 (76) | 17 (62) | 0.19 |
| SAPS II | 66 (44;81) | 71 (56;82) | 0.34 |
| BMI*,* kg/m^2^ | 25 (23;29) | 29 (25;38) | 0.003 |
| BMI<22kg/m^2^ | 14 (18) | 2 (7) | 0.19 |
| At inclusion |  |  |  |
| SOFA score | 15 (12;18) | 15 (12;19) | 0.78 |
| Weight, kg | 80 (71;90) | 95 (71;106) | 0.07 |
| Height, meter | 1.73 (1.66-1.78) | 1.72 (1.61-1.76) | 0.26 |
| ICU admission-to-ECMO interval, d | 1 (0;5) | 4 (0;11) | 0.11 |
| ICU admission-to-C_max_ interval, d | 8 (5;15) | 10 (7;16) | 0.73 |
| ECMO-to-C_max_ interval, d | 6 (3;12) | 6 (3;9) | 0.73 |
| Inotrope score, μg/kg/min | 34 (6;140) | 16 (5;88) | 0.39 |
| Reason for ECMO |  |  | 0.81 |
| Cardiogenic shock | 36 (46) | 13 (48) |  |
| Post-cardiac transplant | 7 (9) | 2 (7) |  |
| Cardiac arrest | 4 (5) | 3 (1) |  |
| Post-cardiotomy | 5 (6) | 1 (4) |  |
| Severe ARDS | 27 (34) | 8 (30) |  |
| VA-ECMO | 53 (67) | 19 (70) | 0.75 |
| ECMO flow, L/min | 4.0 (3.2;5.0) | 3.5 (2.8;4.7) | 0.003 |
| Weight-Indexed ECMO flow, L/min/kg | 0.50 (0.41-0.61) | 0.41 (0.32-0.56) | 0.012 |
| ECMO-membrane duration, d | 5 (2;8) | 6 (2;9) | 0.83 |
| Laboratory finding |  |  |  |
| Aspartate aminotransferase, mmol/L | 71 (41;267) | 85 (48;263) | 0.83 |
| Alanine aminotransferase, mmol/L | 54 (26;155) | 83 (31;231) | 0.97 |
| Bilirubin, mmol/L | 27 (17;66) | 27 (12;66) | 0.38 |
| Prothrombin time, % | 64 (50;75) | 69 (58;83) | 0.95 |
| V factor, % | 76 (46;117) | 69 (45;95) | 0.76 |
| Proteinemia, g/L | 52 (46;57) | 55 (43;60) | 0.42 |
| Albuminemia, g/L | 21 (19;25) | 21 (18;25) | 0.10 |
| Prealbuminemia, g/L | 0.13 (0.09;0.18) | 0.13 (0.09;0.21) | 0.56 |
| Hematocrit, % | 24 (22;27) | 26 (25;29) | 0.04 |
| Lactates, mmol/L | 1.9 (1.2;4.8) | 1.8 (1.3;3.3) | 0.68 |
| Hemodilution parameter |  |  |  |
| 24-h fluid balance, mL | 321 (-831;1828) | –182 (–1090;1461) | 0.35 |
| 24-h protidemia delta, % | 0 (–5.2;3.0) | 0 (–4.8;4.4) | 0.79 |
| 24-h hematocrit delta, % | -1.8 (–9.3;6.6) | 0.0 (–8.5;9.6) | 0.08 |
| GFR, mL/min | 12 (0;71) | 0 (0;45) | 0.80 |
| Renal function |  |  | 0.26 |
| KDIGO-0 | 26 (33) | 4 (15) |  |
| KDIGO-1 | 8 (10) | 3 (11) |  |
| KDIGO-2 | 5 (6) | 1 (4) |  |
| KDIGO-3 | 40 (51) | 19 (70) |  |
| KDIGO ≥2 | 45 (57) | 20 (74) |  |
| Dialysis | 4 (5) | 5 (18) | 0.03 |
| CRRT | 34 (43) | 11 (41) | 0.83 |
| Outcome |  |  |  |
| ICU mortality | 41 (52) | 16 (59) | 0.32 |
| Hospital mortality | 42 (53) | 16 (59) | 0.58 |
| ECMO duration, d | 17.0 (8.2;26.0) | 19 (11;26) | 0.36 |
| Mechanical ventilation duration, d | 22 (12;40) | 27 (14;46) | 0.39 |
| RRT duration after C_max_, d | 13 (4;22) | 19 (8;24) | 0.28 |
| AKI^b^ at ICU discharge for survivors^c^ | 9/38 (24) | 2/11 (18) | 0.70 |

## AKI = acute kidney injury; ARDS = acute respiratory distress syndrome, BMI = body mass index, CRRT = continuous renal replacement therapy, GFR = glomerular filtration rate, ICU = intensive care unit, KDIGO = Kidney Disease: Improving Global Outcomes, SAPS = Simplified Acute Physiology Score, SOFA = Sequential Organ-Failure Assessment, VA-ECMO = venoarterial extracorporeal membrane oxygenation.

## ^a^Values are expressed as median (1^st^;3^rd^ quartile]) or *n* (%).

^b^Defined as KDIGO ≥ 2.

^c^Based on 49 ICU survivors

**Additional file 5: Amikacin C_max_ distribution within our population on ECMO with 25mg/kg for all patients or with using adapted dosing regimens based on 24-hour fluid balance and BMI, as described in Table 3.**

**
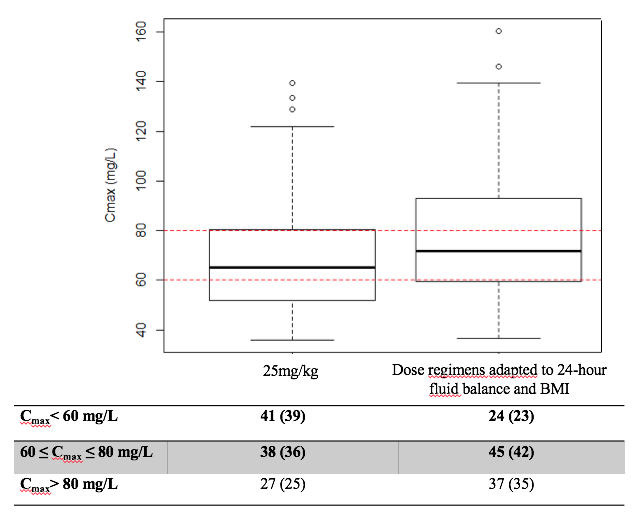
**
